# Patient Characteristics, Management, and Outcomes in a Novel Cohort of Primary Hyperparathyroidism

**DOI:** 10.1210/jendso/bvae096

**Published:** 2024-05-09

**Authors:** Vivek R Sant, Yaser ElNakieb, Christoph U Lehmann, Justin F Rousseau, Naim M Maalouf

**Affiliations:** Division of Endocrine Surgery, UT Southwestern Medical Center, Dallas, TX 75390, USA; Clinical Informatics Center, UT Southwestern Medical Center, Dallas, TX, 75390, USA; Clinical Informatics Center, UT Southwestern Medical Center, Dallas, TX, 75390, USA; Department of Neurology, UT Southwestern Medical Center, Dallas, TX 75390, USA; Peter O’Donnell Jr. Brain Institute, UT Southwestern Medical Center, Dallas TX, 75390, USA; Department of Internal Medicine and Charles and Jane Pak, Center for Mineral Metabolism and Clinical Research, UT Southwestern Medical Center, Dallas, TX 75390, USA

**Keywords:** primary hyperparathyroidism, parathyroidectomy, hypercalcemia, fracture, chronic kidney disease, TriNetX

## Abstract

**Context:**

Primary hyperparathyroidism (PHPT) increases the risk of bone loss, debilitating fractures, kidney stones, impaired renal function, and neurocognitive symptoms. Studies describing the natural history of PHPT have been limited to small samples, single institutions, or specific populations.

**Objective:**

We assessed the natural history of PHPT through a large, diverse national cohort from an electronic health record dataset representing more than 100 million patients.

**Methods:**

The TriNetX database was queried for adult patients with PHPT. We extracted demographics, comorbidities, and longitudinal biochemistries. Primary outcomes included major osteoporotic fracture (MOF) and chronic kidney disease (CKD). Outcomes were stratified by treatment strategy (surgical parathyroidectomy [PTX] vs nonsurgical) and age.

**Results:**

Among 50 958 patients with PHPT, 26.5% were treated surgically at a median of 0.3 years postdiagnosis. At diagnosis, median age was 65 years, 74.0% were female, and median calcium level was 10.9 mg/dL. Black and older patients underwent PTX less frequently than White and younger patients. MOF 10-year incidence was 5.20% (PTX) and 7.91% (nonsurgical), with median 1.7-year delay with PTX compared to nonsurgical. PTX-associated MOF absolute risk reduction was 0.83% (age < 65 years) and 3.33% (age ≥ 65 years). CKD 10-year incidence was 21.2% (PTX) and 33.6% (nonsurgical), with median 1.9-year delay with PTX. PTX-associated CKD absolute risk reduction was 12.2% (age < 65 years) and 9.5% (age ≥ 65 years).

**Conclusion:**

We report 1 of the largest, representative, population-based natural histories of PHPT with different management strategies. A minority of patients underwent PTX, especially in older age. Patients managed surgically had lower incidence of fracture and CKD, and older patients experienced differential benefit.

Primary hyperparathyroidism (PHPT) is the third most common endocrine disorder, with a prevalence of 1.6 in 1000 Americans. PHPT disproportionately affects patients of advanced age, female sex, and Black race [1, 2]. Patients are at increased risk of bone loss, debilitating fractures, kidney stones, impaired renal function, and neurocognitive symptoms. Surgical management, through parathyroidectomy (PTX), is currently the only feasible and durable cure. The American Association of Endocrine Surgeons and the Fifth International Workshop on Evaluation and Management of Primary Hyperparathyroidism have published clinical practice guidelines with concordant and well-accepted minimum criteria for recommending surgical management [3, 4]. However, guideline-concordant surgical management only occurs in only 40% of eligible patients [5, 6].

To understand the discrepancy between guideline recommendation and practice, studies analyzed the epidemiology and natural history of PHPT as well as the differences in patient and disease factors associated with guideline-concordant care. Studies in Rochester, MN, and at Kaiser Permanente first illuminated epidemiology and natural history of PHPT among large population-based cohorts but were limited to specific geography or a single vertically integrated health care system [1, 2]. A study of the Veterans Affairs Health Care System shed light on underdiagnosis of hyperparathyroidism but was limited to the veteran population comprising 90% men in a disease predominantly occurring in women [7]. Studies of the Medicare population further elucidated differences in complication risks between surgical and nonsurgical cohorts but were limited to the 65+ year Medicare population and were constrained by the lack of access to laboratory data required to assess disease severity and extent of complications such as chronic kidney disease (CKD) [5, 8].

To overcome the limitations of prior datasets, we identified a large and diverse national cohort of patients with PHPT from an electronic health record dataset (TriNetX) [9] with more than 100 million patients, to characterize this population better, and to identify the respective rates of complications among surgical (PTX) and nonsurgical groups. We hypothesized that our cohort would be congruent with previously reported national demographics of PHPT, rates of disease-related complications, and surgical management choices, whereas being more representative of the overall population and provide important additional data such as laboratory test results.

## Materials and Methods

### Data Sources

We used the TriNetX dataset, which includes electronic health record data representing 100 million patients across 83 health care organizations throughout the United States and additional countries. We queried the dataset in January 2024 to identify patients with PHPT.

### Cohort Construction

**Figure 1.**
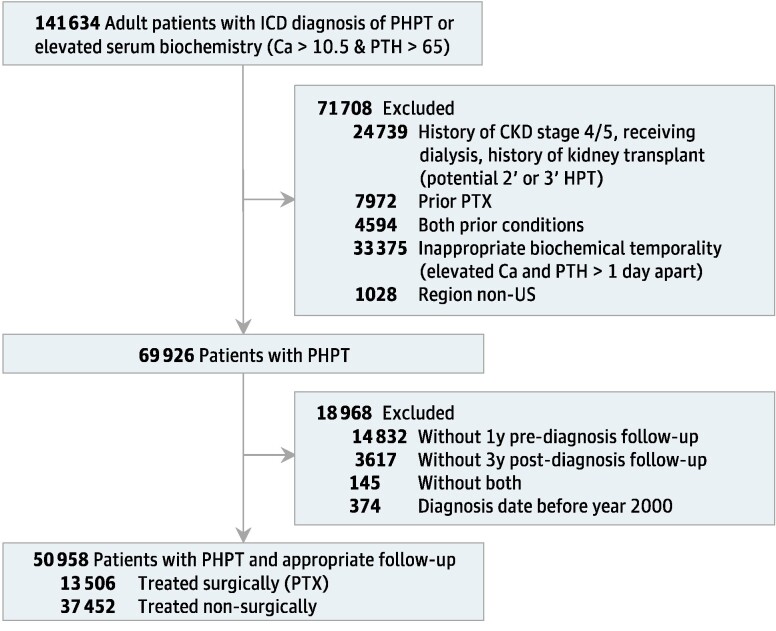
Study cohort enrollment.

Our flow diagram of cohort construction is shown in Fig. 1. The query was limited to adult patients (aged 18 years or older). Patients were included from time of the first diagnosis of PHPT by International Classification of Diseases (ICD) code (ICD-10 E21.0 with automated ICD-9 conversion by TriNetX) or serum biochemistry (first episode of jointly elevated calcium [>10.5 mg/dL] and PTH [>65 pg/mL] within 1 day of each other). We excluded patients who were potentially miscoded with underlying secondary or tertiary hyperparathyroidism—by history of CKD stage 4 or 5, actively receiving dialysis, or history of kidney transplant before PHPT diagnosis.

To account for inaccurate coding or confounding from recurrent or persistent PHPT, patients with history of PTX before PHPT diagnosis date were also excluded. Patients with a “region” code indicating that they lived outside of the United States were also excluded with the belief that PHPT management within a national cohort would be more likely to be homogeneous. To account for progressive historical adoption of surgical guidelines for asymptomatic PHPT, bone mineral density testing, and bisphosphonate treatment of osteoporosis, patients diagnosed with PHPT before 2000 were excluded. Finally, to ensure appropriate entry into the cohort, patients were excluded if they did not have 1 year of records before index date, to ensure that PHPT and other diagnoses were not historical entries being recorded on a patient's first encounter with a health care system. To ensure appropriate follow-up, patients were also excluded if they did not have 3 years of follow-up, as identified by the presence of inpatient or outpatient encounters.

### Outcomes

Primary outcomes of interest included major osteoporotic fractures ([MOF]; hip, spine, wrist, and humerus) events and development of CKD. Diagnoses were identified by ICD and Current Procedural Terminology codes [10]. The outcomes were identified at discrete time points following initial PHPT diagnosis and reported in graphical format as a cumulative incidence curve. The outcomes were subsequently stratified by age: <65 and ≥65 years. Fracture outcomes were also stratified by initial bone mineral density diagnosis as identified by ICD code.

### Variables

Variables extracted included patient demographics (age, gender, race/ethnicity, height, weight, body mass index, geographic region), comorbidities by diagnosis codes, and serum biochemistry over time (calcium and PTH). We identified the receipt of PTX during follow-up using Current Procedural Terminology codes (60500, 60502). Calcium values were reported at several individual time points, including closest to time of diagnosis (cohort inclusion index). For patients who underwent PTX, additional time points were reported for calcium levels including highest value before PTX and first level at least 6 months after PTX (the accepted timepoint post-PTX to determine cure from PHPT) [3].

### Analysis

We described the whole cohort and subsequently stratified by those who underwent PTX (surgical management) and those who did not (nonsurgical management). Univariate statistical analysis was performed to describe the cohort as a whole. Within the stratified groups, we described binary variables through counts and percentages and continuous variables through median values and interquartile ranges (IQR). We presented the cumulative incidence for outcomes on curves, with the starting time point for the nonsurgical group at time of cohort inclusion by diagnosis and the starting time point for the surgical group at the time of surgery. Outcome incidence curves were compared using log-rank test with Peto modification to improve power for expected delayed censoring. We also recorded the number of patients present at discrete time intervals of follow up.

## Results

### Cohort Description

Our final cohort selection yielded 50 958 patients, including 13 506 treated surgically and 37 452 treated nonsurgically. Table 1 summarizes the characteristics of the cohorts overall and stratified by treatment modality. The overall cohort had a median age of 65 years at diagnosis, 14.3% of patients were aged 50 years or younger, 30.5% between ages 66 and 75 years, and 18.6% above age 75 years. Most patients (74.0%) were female and White (66.9%). All US geographic regions were well represented, including South, Northeast, Midwest, and West. Surgical and nonsurgical groups had a few notable differences. The nonsurgical group had a higher percentage of patients older than age 75 years (21.7%) compared to the surgical group (10.1%). White race comprised a greater proportion of the surgical group (72.4%) compared to nonsurgical (64.9%), whereas Black race comprised a greater proportion of the nonsurgical group (20.0%) compared to surgical (13.2%). Geographic regions were similarly represented in both strata, as were anthropometric measures of height, weight, and body mass index. Differences in rates of surgical management by demographic strata are further described in Table 2. Surgical management was used in 25.2% of those aged 66 to 75 years, and in 14.4% with age > 75 years. Among White patients, 28.7% were managed surgically; 19.2% of Black patients were managed surgically.

**Table 1.** Characteristics of cohort

Characteristic	Overall	Parathyroidectomy	Nonsurgical
Age, median (IQR)	65.00 (56.00-73.00)	62.00 (54.00-70.00)	66.00 (57.00-74.00)
Age, y			
≤ 50	7284 (14.3%)	2354 (17.4%)	4930 (13.2%)
51-65	18 650 (36.6%)	5867 (43.4%)	12 783 (34.1%)
66-75	15 522 (30.5%)	3916 (29.0%)	11 606 (31.0%)
>75	9502 (18.6%)	1369 (10.1%)	8133 (21.7%)
Gender			
Female	37 713 (74.0%)	10 390 (76.9%)	27 323 (73.0%)
Male	11 793 (23.1%)	2902 (21.5%)	8891 (23.7%)
Race/ethnicity			
Race—Black or African American	9277 (18.2%)	1781 (13.2%)	7496 (20.0%)
Race—White	34 085 (66.9%)	9775 (72.4%)	24 310 (64.9%)
Race—Asian, American Indian, Pacific Islander, Other/unknown	7596 (14.9%)	1950 (14.4%)	5646 (15.1%)
Ethnicity—Hispanic or Latino	2016 (4.0%)	601 (4.4%)	1415 (3.8%)
Region			
South	16 365 (32.1%)	3867 (28.6%)	12 498 (33.4%)
West	5842 (11.5%)	1880 (13.9%)	3962 (10.6%)
Midwest	12 910 (25.3%)	3895 (28.8%)	9015 (24.1%)
Northeast	15 841 (31.1%)	3864 (28.6%)	11 977 (32.0%)
Anthropometrics			
Weight, kg: median (IQR)	79.80 (66.68-95.10)	81.01 (68.04-96.03)	79.30 (66.22-94.62)
Height, cm: median (IQR)	165.10 (158.75-170.99)	165.10 (160.00-171.50)	165.10 (157.78-170.20)
BMI: median (IQR)	29.00 (25.00-33.78)	29.20 (25.30-33.98)	28.89 (24.89-33.69)
Bone density diagnoses			
Osteopenia	3904 (7.7%)	1511 (11.2%)	2393 (6.4%)
Osteoporosis	7475 (14.7%)	2747 (20.3%)	4728 (12.6%)
Complications of PHPT at time of diagnosis			
Fracture history at PHPT diagnosis	1300 (2.55%)	279 (2.07%)	1021 (2.73%)
Nephrolithiasis history at PHPT diagnosis	4796 (9.41%)	1640 (12.14%)	3156 (8.43%)
Stage 3 CKD history at PHPT diagnosis	7537 (14.79%)	1091 (8.08%)	6446 (17.21%)

Abbreviations: BMI, body mass index; CKD, chronic kidney disease; IQR, interquartile range; PHPT, primary hyperparathyroidism.

**Table 2.** Breakdown of patients undergoing PTX by demographic characteristics

Characteristic	Proportion by strata undergoing PTX
Age, y	
≤ 50	32.3%
51-65	31.5%
66-75	25.2%
>75	14.4%
Gender	
Female	27.6%
Male	24.6%
Race/ethnicity	
Race—Black or African American	19.2%
Race—White	28.7%
Race—Asian, American Indian, Pacific Islander, Other/unknown	25.7%
Ethnicity—Hispanic or Latino	29.8%
Region	
South	23.6%
West	32.2%
Midwest	30.2%
Northeast	24.4%

Abbreviation: PTX, parathyroidectomy.

### Cohort Inclusion by Biochemistry and Diagnosis Code

Inclusion in the cohort was largely through PHPT diagnosis code AND biochemistry (33.2%), along with diagnosis code alone (40.3%), and biochemistry alone (26.5%). Of the patients who underwent PTX, 93.8% met inclusion by diagnosis code.

### Biochemical Assessment

Biochemical findings are described in Table 3. The overall cohort had a median calcium of 10.9 mg/dL and PTH of 104.6 pg/mL. Median calcium was 10.9 mg/dL in the surgical group and 10.8 mg/dL in the nonsurgical group. Within the surgical group, the median highest calcium before surgery was 11.2 mg/dL and median calcium 6 months postoperatively was 9.4 mg/dL.

**Table 3.** Biochemical distribution of cohort

Serum labs	Overall	Parathyroidectomy	Nonsurgical
Closest to time of diagnosis			
Ca, median (IQR), mg/dL	10.9 (10.7-11.1)	10.9 (10.7-11.2)	10.8 (10.7-11.1)
PTH, median (IQR), pg/mL	104.6 (81.6-148.2)	111.4 (86.0-155.6)	102.0 (80.0-144.7)
Highest Ca before PTX, median (IQR), mg/dL	N/A	11.2 (10.8-11.6)	N/A
Ca ≥ 6 mo after PTX, median (IQR), mg/dL	N/A	9.4 (9.1-9.7)	N/A

Abbreviations: Ca, calcium; IQR, interquartile range; N/A, not available.

### Chronology and Follow-up

In patients with PTX, the time between diagnosis (by ICD code or biochemistry) and surgery was a median of 0.3 years (IQR 0.1-1.3). Follow-up data were present in 32 033 (62.9%) patients at 5 years and 8752 (17.2%) at 10 years.

### Primary Outcomes

**Figure 2.**
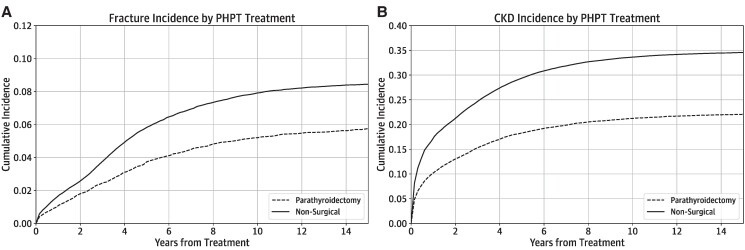
Cumulative incidence of PHPT outcomes including (A) fracture and (B) CKD.

Figure 2 details the cumulative incidence of outcomes in the cohort stratified by treatment modality. Outcome incidence is also reported in tabular form [10]. Cumulative incidence of MOF in the overall cohort was 3.38% at 3 years, 5.28% at 5 years, and 7.35% at 10 years, with median time to fracture 4.3 years (IQR 2.4-7.1 years). CKD was present in 22.2%, 26.6%, and 30.8% of the cohort at 3, 5, and 10 years, respectively, with incidence at a median time of 3.8 years (IQR 1.9-6.5 years). Fracture and CKD incidence curves diverged by PHPT treatment modality, with higher incidences of fractures (*P* = .016) and CKD in the nonsurgical group (*P* < .001).

### Age-stratified Outcomes

**Figure 3.**
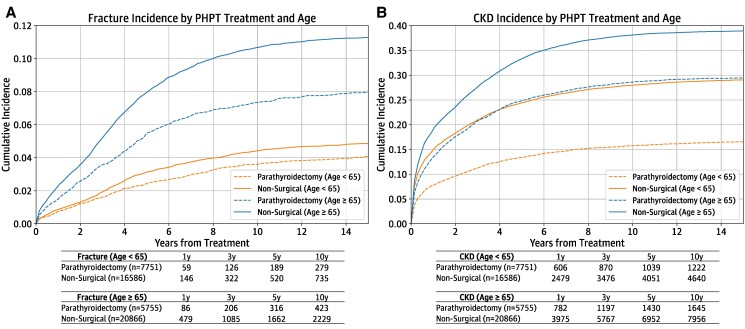
Age-stratified incidence of (A) fracture and (B) CKD.

Cumulative incidence curves are detailed in Fig. 3, stratifying fracture and CKD outcomes by age threshold of 65 years. The cumulative incidence of fracture was higher in the age ≥ 65-year group, with PTX reducing fracture incidence in both age groups. In the < 65-year age group, 10-year cumulative fracture incidence was 3.60% in the PTX group and 4.43% in the nonsurgical group (absolute risk reduction [ARR] 0.83%), and in the ≥ 65-year age group, 7.35% and 10.68%, respectively (ARR 3.33%). Cumulative incidence of CKD for both age groups similarly diverged by age. CKD incidence was lower with PTX in both age groups. In the < 65-year age group, 10-year cumulative CKD incidence was 15.8% in the PTX group and 28.0% in the nonsurgical group (ARR 12.2%), and in the ≥ 65-year age group, 28.6% and 38.1%, respectively (ARR 9.5%).

### Bone Density-stratified Fracture Outcomes

**Figure 4.**
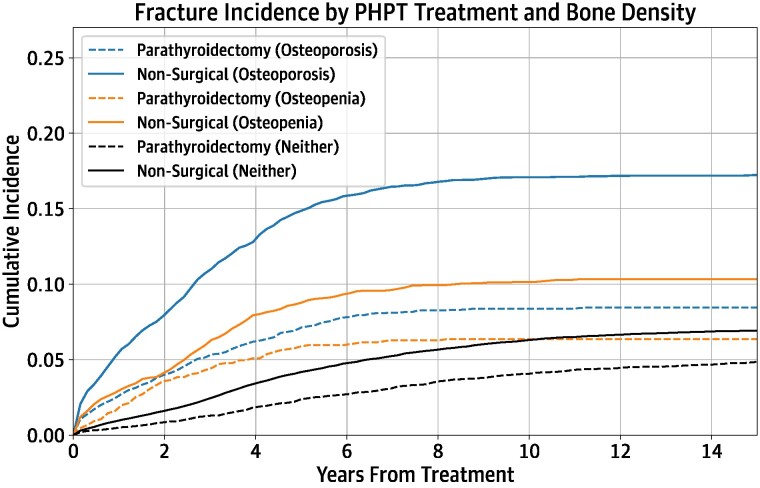
Bone density-stratified incidence of fracture.

Cumulative incidence curves are detailed in Fig. 4, stratifying fracture outcomes by bone density diagnosis. The cumulative incidence of fracture was highest in the group with osteoporosis, followed by the group with osteopenia. Fracture risk was lower in the PTX group compared to the nonsurgical group, in all 3 bone density strata. In the osteoporosis group, 10-year cumulative fracture incidence was 8.37% in the PTX group and 17.09% in the nonsurgical group (ARR 8.72%). In the osteopenia group, 10-year cumulative fracture incidence was 6.35% in the PTX group and 10.15% in the nonsurgical group (ARR 3.80%). In patients with neither diagnosis, fracture 10-year cumulative fracture incidence was 4.07% in the PTX group and 6.31% in the nonsurgical group (ARR 2.24%).

## Discussion

We describe a novel, diverse cohort of 50 958 adult patients with PHPT, drawn from across the United States. Our patients with PHPT were primarily older women, of White race, in line with previous reports [1, 11]. A minority (26.5%) underwent surgical management, despite the fact that at least 28.4% of the nonsurgical group already had PHPT-associated complications (fracture, kidney stones, CKD) at the time of diagnosis. Biochemical data were available longitudinally, providing an additional marker of disease severity, and showed normalization postparathyroidectomy, suggesting surgical cure. Finally, our described outcomes in PHPT in this cohort had long-term follow-up and were congruent with previous reports, including lower incidence of fracture and development of CKD in those managed surgically [8, 12-15]. PTX was associated with greater fracture ARR in the older age stratum (age ≥ 65 years), and greater CKD ARR in the younger age stratum (age < 65 years). Our findings are in line with prior efforts, provide new insights regarding surgical benefits in general and age-related differential benefit to PTX, and offer a large, detailed, and more representative cohort than previous reports, which can serve as a foundation for future research.

Past research noted that PHPT manifests primarily in older women [1, 11]. Our cohort is similarly representative with 74.0% women, a median age 65 years, and 18.6% of patients older than 75 years of age. However, 14.3% of patients were age 50 years or younger, and 36.6% ages 51 to 65 years. This age distribution allowed for a more appropriate representation of the full spectrum of patients who may develop and present with PHPT, compared to reported cohorts limited to an age 65 years and older population [11]. Racial breakdown of the cohort was congruent with prior reports and is reflective of the US census [1, 16]. However, 28.7% of White patients were treated surgically, compared to only 19.2% of Black patients. This disparity is similar to prior reports, bears further investigation in future work, and may be addressed through clinical decision support [17, 18]. Numerous reports have highlighted the underutilization of surgical management in PHPT with rates of surgical management ranging from 10% to 40% in various PHPT cohorts. Further disparities in rates of surgical management by race and age remain [5-7, 11, 19, 20]. Our study identifies similar findings of racial disparity in management of PHPT.

By age, additional disparities in surgical management were noted, with only 14.4% older than age 75 years treated surgically. The geriatric patient certainly requires careful consideration of the risks and benefits of surgery, but we report these discrepancies despite the fact that prior work and this report has suggested significant PTX benefit in many patients of advanced age [8]. At the other end of the age spectrum, only 32.3% of patients aged 50 years or younger were treated surgically, despite that age ≤ 50 years is an indication for surgery by consensus guidelines since 1990 and this indication has been confirmed repeatedly through 2022 [4, 21-24]. Additionally, 28.4% of nonsurgical patients already had diagnosis-based indications for surgery (history of fracture, kidney stones, or CKD) at PHPT diagnosis but were still treated nonsurgically throughout follow-up.

Our description of outcomes in PHPT is concordant with previous reports in the literature and provide additional insight on age-specific benefit of PTX. Studies in a national Danish cohort and the vertically integrated Kaiser Permanente system noted significant decrease in fracture at hip and all sites in patients with PTX versus nonsurgical cohorts [12, 13]. A study of a Medicare cohort noted a 10-year ARR of 2.3% for hip fracture and 5.1% for all fractures with PTX [8]. Our own finding of 2.7% ARR with PTX for MOF includes hip fracture but not all fractures. This finding may suggest a potentially lower benefit of PTX at other MOF sites other than the hip, but more likely reflects the inclusion of younger patients, who have lower baseline fracture risk than the older Medicare cohort. Further, older patients with PHPT experienced a greater ARR benefit of MOF with PTX than younger patients perhaps reflecting a threshold effect whereby age-related bone loss coupled with PHPT-related bone loss may accumulate to the greatest risk for older patients with PHPT. In keeping with prior reports [12, 25], patients of all bone density diagnoses (osteoporosis, osteopenia, neither) experienced fracture risk reduction with parathyroidectomy, with the greatest ARR benefit seen in patients with osteoporosis.

Several studies have shown a slowing in renal function decline after PTX compared to nonsurgical management [14, 15]. More recently, a Veterans Administration cohort described no difference in renal decline regardless of modality of management of PHPT [26]. Our findings, although unable to establish causality, note a decreased incidence of declining kidney function with PTX, congruent with earlier studies. Interestingly, younger patients undergoing PTX experienced greater ARR in CKD incidence compared to older patients, reflecting yet another opportunity and potential benefit for early identification and surgical intervention in PHPT.

### Limitations

Our study has some limitations that must be discussed. Although TriNetX covers a large number of health care organizations, patients may still receive care at times at other institutions that may not participate; thus, reported diagnoses and outcomes may be an underestimate of the true prevalence. We expect this to be a limitation of similar magnitude for any study outside of a vertically integrated health care system or claims database. As with any retrospective cohort study, our study is limited by the same concerns of coding accuracy and retrospective bias. Given the lack of an ICD code for familial hypocalciuric hypercalcemia, we are unable to identify patients with overlapping diagnoses who may appropriately not be undergoing surgery; however, given much lower population prevalence of familial hypocalciuric hypercalcemia compared to PHPT, we expect this concern to be of limited magnitude. Also, in our effort to provide a strict cohort definition to minimize false-positive inclusions, we may potentially have missed including patients with more subtle disease such as normocalcemic PHPT.

## Conclusion

Our work provides one of the largest, most representative, and detailed, population-based natural history descriptions of PHPT comparing different management strategies, building on the strengths and improving upon weaknesses of prior work. Patients managed surgically have lower cumulative incidence of fracture and CKD, and older patients experienced differential benefit. This carefully defined cohort with its large size, representative patient population, and rich longitudinal dataset including serum biochemistry may prove valuable for use in future studies.

## Data Availability

Some or all datasets generated during and/or analyzed during the current study are not publicly available but are available from the corresponding author on reasonable request.

## Abbreviations

ARR: absolute risk reduction
CKD: chronic kidney disease
ICD: International Classification of Diseases
IQR: interquartile range
MOF: major osteoporotic fracture
PHPT: primary hyperparathyroidism
PTX: parathyroidectomy
